# Efficient Background Segmentation and Seed Point Generation for a Single-Shot Stereo System

**DOI:** 10.3390/s17122782

**Published:** 2017-12-01

**Authors:** Xiao Yang, Xiaobo Chen, Juntong Xi

**Affiliations:** 1 School of Mechanical Engineering, Shanghai Jiao Tong University, Shanghai 200240, China; yangxiao1992@sjtu.edu.cn (X.Y.); xiaoboc@sjtu.edu.cn (X.C.); 2 Shanghai Key Laboratory of Advanced Manufacturing Environment, Shanghai 200030, China; 3 State Key Laboratory of Mechanical System and Vibration, Shanghai 200240, China

**Keywords:** single-shot 3D measurement, digital image correlation, background segmentation, seed point generation

## Abstract

Single-shot stereo 3D shape measurement is becoming more popular due to its advantages of noise robustness and short acquisition period. One of the key problems is stereo matching, which is related to the efficiency of background segmentation and seed point generation, etc. In this paper, a more efficient and automated matching algorithm based on digital image correlation (DIC) is proposed. The standard deviation of image gradients and an adaptive threshold are employed to segment the background. Scale-invariant feature transform (SIFT)-based feature matching and two-dimensional triangulation are combined to estimate accurate initial parameters for seed point generation. The efficiency of background segmentation and seed point generation, as well as the measuring precision, are evaluated by experimental simulation and real tests. Experimental results show that the average segmentation time for an image with a resolution of 1280 × 960 pixels is 240 milliseconds. The efficiency of seed point generation is verified to be high with different convergence criteria.

## 1. Introduction

3D shape measurement is a significant method to study the morphology characteristics of measuring objects, which has widespread applications in industrial, biomedicine, architectural fields, etc. Optical 3D measurement has achieved tremendous development due to the properties such as non-contact, high precision, high measuring speed, and full-field measurement. Some notable applications include machining precision detection [1], 3D reconstruction [2,3,4,5], vehicle autonomous navigation [6], 3D shape measurement [7,8], indoor mapping [9], etc.

Stereo vision is one of the most commonly used optical 3D techniques, which can be classified into single-shot and multiple-shot methods. Multiple-shot methods are sensitive to vibrational noise, while single-shot methods are more robust and can be employed for real-time measurement. An important advantage of single-shot methods is that there are no synchronization problems between the projector and camera(s). When the measuring object is moving, single-shot methods must be employed. However, multiple-shot methods can achieve higher precision than single-shot methods. Some other drawbacks of single-shot methods are the requirements of precise calibration (to compensate for the tradeoff of precision and baseline length), no repetitive surface texture for stereo correspondence, and computation complexity [10]. In recent decades, the digital image correlation (DIC)-based stereo measurement method has become one of the most popular single-shot methods [11,12,13,14]. For a stereo system, DIC matches the correspondence points in the reference image and target image to obtain a dense disparity map. One of the key problems in stereo DIC is the matching efficiency, which mainly relates to the initial parameters estimation and iteration speed. In order to start full-field measurement, the initial estimation of parameters for the seed point must be reasonably close to the optimized parameters. In the traditional method, the initial estimation is provided by integer displacement searching [15], which only has an accuracy of 0.5 pixels. Pan et al. [16] used feature matching and affine transformation to provide an initial estimation for the seed point. The estimation is accurate, but only one seed point can be generated, which is not suitable for discontinuous shape measurement. Kieu et al. [12] proposed an algorithm that can generate enough seed points to measure separate objects automatically. However, they did not consider the wrong matches, this can lead to a significant number of wrong estimations. The iteration speed is related to the iterative method. Recently, the inverse compositional Gauss-Newton (IC-GN) algorithm is commonly used in DIC because it is equivalent to the classic forward additive Gauss-Newton (FA-GN) method, but it is more efficient [14,15,17,18,19]. The matching efficiency can be further improved with the help of a fast recursive scheme [20], improved initial parameters transfer [15], and reliability-guided seed point propagation [21]. Beyond all that, a considerable factor that affects the efficiency is redundant calculation in the unmatchable region. Conventionally, a region of interest (ROI) is usually pre-set in the reference image before the DIC process [22]. This is not an automatic process and not suitable for 3D shape measurement. Kieu et al. [12] directly used the convergence to determine the unmatchable regions, like the background and shadows, which is not an efficient method. Sum et al. [23] proposed a gradient-based edge detection method to segment the background. However, their method requires that the reference image to be captured twice with two different background colors.

In this work, we present an efficient background segmentation and seed point generation method for a DIC-based single-shot stereo system. The proposed method includes three major steps. In the first step, the standard deviation of gradients and an adaptive threshold are employed to segment the background efficiently. In the second step, scale-invariant feature transform (SIFT)-based feature matching [24], feature points triangulation, and affine transformation are combined to estimate accurate initial parameters for seed point generation. Meanwhile, wrong matches are removed by a two-step method. In the third step, the optimized parameters are transferred to neighboring points as initial parameters to obtain dense correspondences.

The remainder of this paper is organized as follows: The working principle of the single-shot stereo system is introduced in Section 2. In Section 3, we present the process of the proposed stereo matching algorithm. Experimental results and discussions are reported in Section 4. Finally, conclusions are drawn in Section 5.

## 2. Working Principle of Single-Shot Stereo System

**Figure 1:**
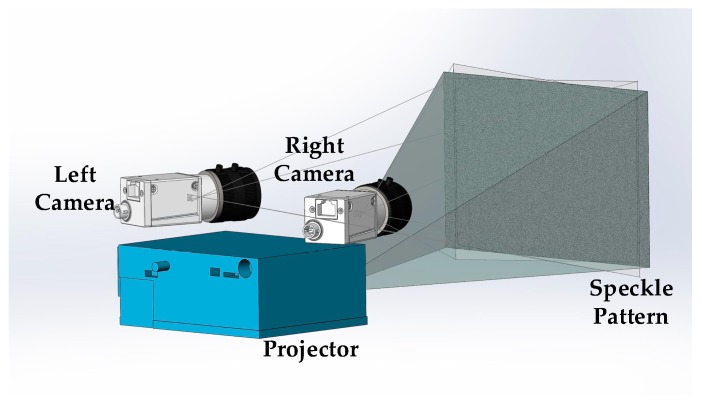
Structure of the single-shot stereo system.

As shown in Figure 1, the single-shot stereo system is composed of two CCD cameras and a digital light projector. As the measuring signal is sent, the projector works immediately, then the left camera and right camera are triggered simultaneously by hardware to do a single-shot, respectively. The projected speckle pattern is to enhance the surface texture or avoid repetitive surface textures that may lead to wrong correspondences.

A whole measuring process includes system calibration [25], stereo image rectification, stereo matching, and 3D reconstruction. Among them stereo matching is the most difficult step, which can be efficiently performed by the proposed algorithm in Section 3.

## 3. Stereo Matching of the Single-Shot Stereo System

### 3.1. Background Segmentation

**Figure 2:**
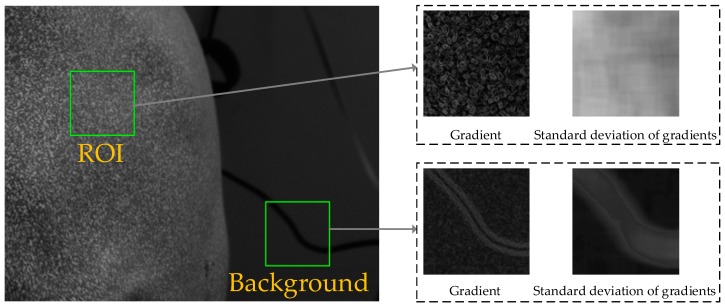
Schematic diagram of background segmentation.

As shown in Figure 2, the background of the captured speckle image is uniform in a small subset, while the ROI is not uniform in a small subset because the intensity of each pixel was randomly generated. In other words, the image gradients of background are uniform in a small subset and not uniform in a small subset of the ROI. Therefore, the standard deviation of gradients in a small subset of the ROI is larger than that of the background. In this paper, a fast and efficient background segmentation method is proposed for the speckle-projected image. The standard deviation of gradients in a small subset is computed by:

(1) $$\sigma \left(x,y\right)=\sqrt{\frac{\sum_{\Delta y=-M}^{\Delta y=M}\sum_{\Delta x=-M}^{\Delta x=M}{\left(g\left(x+\Delta x,y+\Delta y\right)-\bar{g}\right)}^{2}}{{\left(2M+1\right)}^{2}-1}}$$

where g denotes the magnitude of gradients in x direction and y direction. $\bar{g}$ denotes the mean of the g values in the subset. The subset size is (2M+1)×(2M+1). σ denotes the standard deviation of the gradients in the subset. σ can be efficiently calculated for every pixel with the help of the global sum table [20]. We then use the Ostu threshold [26] method to segment the background and ROI.

### 3.2. Feature-Based Matching for the Coarse-Matched Triangle Set

#### 3.2.1. Coarse Match by Scale-Invariant Feature Transform

SIFT is invariant to affine transformation and rotation for detecting local features. According to Lowe’s paper [24], features extracted by SIFT are invariant to image scale, rotation angle, and image luminance. A general process to obtain features includes scale-space extreme detection, keypoint localization, orientation assignment, and keypoint descriptors. For each keypoint, a 128-dimensional vector is established to describe its feature. Then the FLANN [27] algorithm helps to match the feature points, the descriptor of the feature point in the reference image should have the shortest Euler distance to that in the target image.

#### 3.2.2. Removal of Wrong Matches

**Figure 3:**
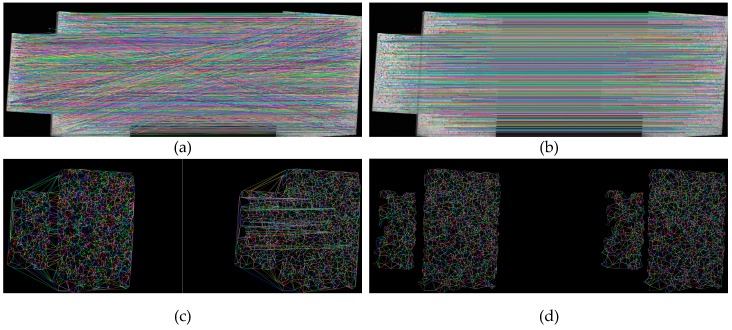
Feature matching by SIFT: (**a**) coarse matches; (**b**) removal of wrong matches by the epipolar line constraint; (**c**) triangulation of matched points in (**b**); and (**d**) removal of wrong matches in (**c**) by setting the area threshold.

After stereo calibration [28], the stereo image pair can be rectified to make the left image and corresponding right image alignment due to the epipolar line constraint. Figure 3 shows the feature matching of a step surface using the proposed method. Figure 3a shows the result of coarse matches of the rectified stereo image pair by SIFT. Since the image pair has been rectified, the matched feature points should lie in the same line. Therefore, the cross lines in Figure 3a mean the wrong matches. In this work, two steps are executed to reduce the wrong matches. In the first step, the maximum absolute difference of the row coordinates of the matched two feature points is set to be 1. Figure 3b shows the matches after removing the point pairs that do not satisfy the alignment requirement. We can see that the cross lines in Figure 3a are almost eliminated. In the second step, the feature points of the left image in Figure 3b are triangulated into a two-dimensional triangular mesh by the Delaunay algorithm [29]. For each triangle in the grid, all three vertices have three corresponding matched points in the right image. Then the three corresponding points are connected into a new triangle in the right image, as Figure 3c shows. The two triangles are denoted as a coarse-matched triangle pair. Then two linked lists are built to store the triangle pairs, namely, left triangle list and right triangle list. Each triangle in the left list corresponds to the one in the right list that at the same position as the left one. There are some intersecting lines in the right image of Figure 3c, which means the matched vertices of some triangles have wrong matches. The left image and right image have the same scale, therefore, the areas of the two corresponding triangles are roughly equal since the vertices of each triangle are assumed to be matched.

(2) $$\frac{Max\left(S_{Left_{i}},S_{Right_{i}}\right)}{Min\left(S_{Left_{i}},S_{Right_{i}}\right)}<\delta ,\;0\leq i<N$$

where N is the size of triangle list. $S_{Left_{i}}$ and $S_{Right_{i}}$ denote the areas of triangles in the left list and right list with index i, respectively. δ denotes the error threshold for the ratio of the larger area to the smaller area. A proper value for δ can be set according to the image resolution and experimental tests, which is set to be 1.2 in this work. The triangle pairs that exceed the area ratio threshold in Equation (2) are removed from the triangle lists. Additionally, the triangle pairs are also removed from the lists if one of the angles of the triangle in the left list is smaller than 20 degrees. Figure 3d shows the result after removing the wrong matches by the two steps. Obviously, the intersecting lines in the right image of Figure 3c are eliminated.

### 3.3. Seed Point Generation and Propagation by IC-GN

Based on the coarse-matched triangle pairs, DIC is employed to obtain the exact correspondence for every pixel to be matched. Firstly, the nearest pixel to each triangle center is chosen to perform DIC to obtain a seed point set. Then, each seed point propagates to its four adjacent pixels with a step size one by one until all the pixels to be matched in the ROI have been propagated.

#### 3.3.1. First-Order DIC Using IC-GN

**Figure 4:**
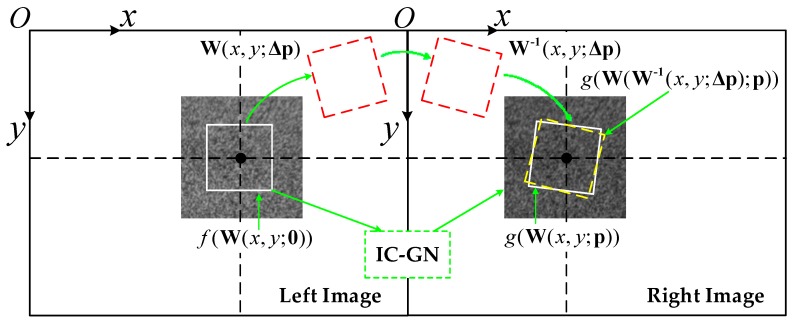
Schematic principle of DIC using the IC-GN algorithm.

Figure 4 shows a schematic process of the application of DIC on a rectified stereo image pair using the first-order IC-GN algorithm. The shape mapping for the reference subset and target subset is described by a first order warp function:

(3) $$W\left(x,y;p\right)=\left[\begin{array}{c} x^{\prime }\\ y^{\prime }\\ 1 \end{array}\right]=\left[\begin{array}{ccc} 1+u_{x} & u_{y} & u\\ v_{x} & 1+v_{y} & v\\ 0 & 0 & 1 \end{array}\right]\left[\begin{array}{c} x\\ y\\ 1 \end{array}\right]$$

(4) $$p={\left(u,u_{x},u_{y},v,v_{x},v_{y}\right)}^{T}$$

where p is the warp parameter vector. u and v are the displacement components of the center pixel in the x direction and y direction, respectively. The other four parameters are the first-order gradient components. In each iteration of DIC, the reference subset and the target subset are compared to solve for the optimal parameter increment vector Δp for next iteration. The process of the IC-GN algorithm can be summarized into three steps. Firstly, compute the increment warp W(x,y;Δp) by exerting Δp on the reference subset. Secondly, invert the increment warp subsequently and compose with the current warp W(x,y;p) to obtain the updated warp:

(5) $$W\left(x,y;p\right)=W^{-1}\left(x,y;\Delta p\right)\cdot W\left(x,y;p\right)$$

Thirdly, repeat the above two steps until the convergence criteria have been met. The iteration speed of IC-GN is about three to five times faster than the FA-NR method because the Hessian matrix remains the same in each iteration [15]. The zero-mean normalized sum of squared difference (ZNSSD) criterion is used to compute the increment warp and Hessian matrix for IC-GN iteration [15]. B-spline interpolation is used to determine the intensity of sub-pixel coordinates on the target subset, which is more accurate than bicubic interpolation [18,30].

#### 3.3.2. Seed Point Generation and Efficient Propagation

For every matched triangle pair obtained in Section 3.2, the nearest pixel to the left triangle center is chosen to perform DIC. The initial values of p can be solved by the six vertices of the triangle pair by affine transformation. If the DIC iteration meets the convergence criteria, the exact disparity of the center pixel can be obtained. The center pixel and the optimized p are added into the seed point set.

**Figure 5:**
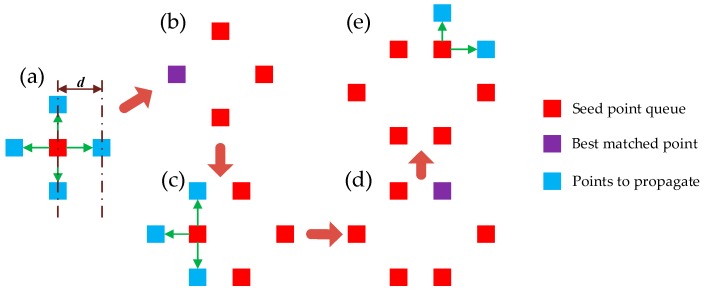
Optimal seed point propagation. (**a**) Propagation of the mother seed point. (**b**) Propagation result of (**a**). (**c**) Propagation of the optimal seed point in (**b**). (**d**) Propagation result of (**c**). (**e**) Propagation of the optimal seed point in (**d**).

Figure 5 shows the process of optimal seed point propagation. The red pixel in Figure 5a denotes the mother seed from the seed point set, the optimized parameters are transferred to the four adjacent blue pixels as initial parameters for DIC. To reduce the computation time, a grid step d is used for the propagation to adjacent pixels. The propagated pixel is inserted into a new ordered seed point queue according to the matching quality if the DIC process succeeds. The seed point with the best matching quality in the queue is considered as the optimal seed [21], which is at the front of the queue. After the optimal seed point has propagated to its four directions, it is removed from the queue. Assuming all the propagations are successful, there are four new seed points in the queue as the blue pixels in Figure 5a. If the purple pixel in Figure 5b is the optimal seed point, it will be the next pixel to be propagated. The pixels that have been propagated successfully will not be propagated again. When the seed point queue becomes empty, the mother seed is removed from the seed point set and the next seed point in the set becomes the new mother seed. The propagation process ends when the seed point set becomes empty or all the presupposed pixels in the ROI have been propagated.

## 4. Experiments and Discussions

**Figure 6:**
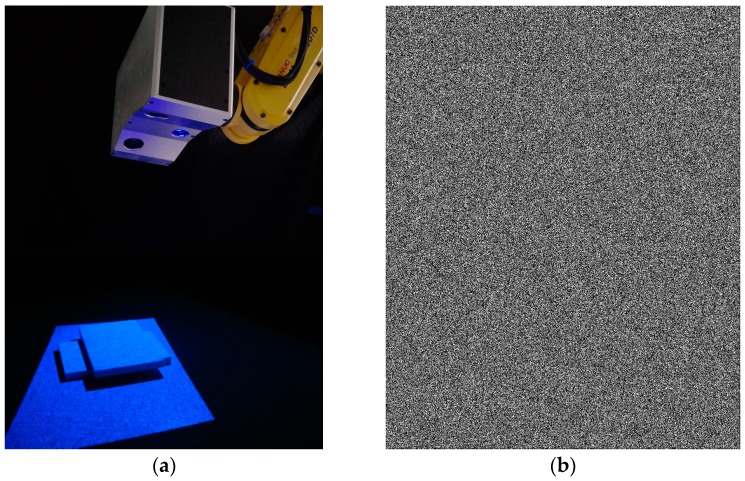
Experimental system. (**a**) Single-shot stereo system with speckle projection; and (**b**) randomly-generated speckle pattern.

The sensor system is composed of two Charge Coupled Device (CCD) cameras (Basler acA1300-30 gm. Manufactured by Basler AG, Ahrensburg, Germany. Supplied by Shanghai Vision-Light tech Co., Ltd. Pudong New Area, Shanghai, China) with a resolution of 1280 × 960 pixels, two camera lenses (Computar 8 mm 1:1.4 2/3. Manufactured by Computar^®^, Tokyo, Japan. Supplied by Shanghai Vision-Light tech Co., Ltd. Pudong New Area, Shanghai, China), and a projector (TI DLP LightCrafter4500. Manufactured by TEXAS INSTRUMENTS, Dallas, Texas, America. Supplied by Texas Instruments Semico…es (Shanghai) Co. Ltd. Pudong New Area, Shanghai, China) with a resolution of 1140 × 912 pixels. As shown in Figure 6a, the sensor is fixed onto a robot end-effector and pre-calibrated by Zhang’s calibration algorithm [31]. Figure 6b shows the speckle pattern, the intensity of each pixel is generated by the sum of some individual Gaussian speckles [32]. There are a total of 30,000 speckles in the gray image with a resolution of 1140 × 912 pixels, and the speckle radius is two pixels.

The speckle pattern is projected onto the surface of the measuring object by the blue channel of the projector. To verify the validation of the proposed method, several experiments are done to test the efficiencies of the background segmentation and seed point generation, as well as to evaluate the measuring accuracy. All the experiments are finished in a laboratory environment and executed by C++ language on a normal Intel(R) Core(TM) i7-4710MQ CPU 2.50 GHz laptop without any acceleration techniques. A larger subset size can achieve higher precision, but leads to much larger calculations. According to Pan’s study [33], the subset size of 21 × 21 is used in this system. In order to study the measurement results more directly, the grid step d for seed point propagation is set to be 1 in our experiments.

### 4.1. Efficiency Test of Background Segmentation

**Figure 7:**
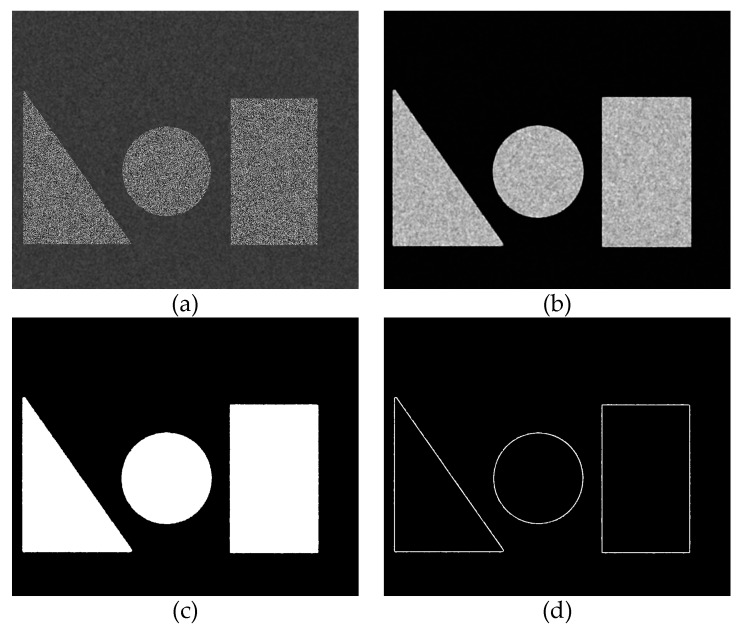
Simulation of the proposed background segmentation method. (**a**) Simulative image; (**b**) σ value map; (**c**) segmentation result; and (**d**) segmentation errors.

Figure 7a shows a simulative image generated by computer with a resolution of 1140 × 912 pixels. The patterns in the triangle, circle, and rectangle regions are the same as in Figure 6b. The intensities of the background are randomly generated between the interval of 0 to 120, and a box filter [34] is used to smooth the background. Figure 7b shows the σ value map computed by Equation (1), while M is 3 here. Figure 7c is the segmentation result of the simulative image and Figure 7d shows the segmentation errors, which are highlighted in white. We can see that the background and ROI are segmented effectively. The errors are all on the edges of the ROI, because the subset of the pixel on the edge includes the pixels in both of the ROI and the background.

**Figure 8:**
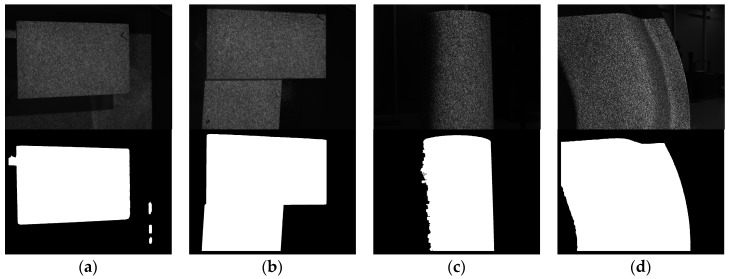
Experimental tests of proposed background segmentation method. (**a**) A plane surface; (**b**) a step surface; (**c**) a cylinder surface; and (**d**) a freeform surface.

Figure 8 shows the real tests of the proposed method. Four captured images are tested, i.e., plane surface, step surface, cylinder surface, and freeform surface. M is set to be 12 in the real tests to reduce the noise in the subset. We can see that the proposed method works well even the illuminations of the four images are different from each other. In Figure 8c, the edge of the segmented ROI is blurry because the edge of the cylinder in the captured image is not distinct. This is not critical because the purpose of segmentation is to reduce unnecessary calculations, which do not need to be accurate. The calculation time for each image is about 240 milliseconds. The calculations of the four images are the same, theoretically, and uncorrelated to the subset size because the use of global sum table.

### 4.2. Efficiency Test of Seed Point Generation

The changes of $\Delta p={\left(\Delta u,\Delta u_{x},\Delta u_{y},\Delta v,\Delta v_{x},\Delta v_{y}\right)}^{T}$ are directly related to the convergence of IC-GN iteration. However, merely Δu and Δv are the determinate components for the final displacements to be determined [17]. Therefore, we use the modulus of Δu and Δv, $||\Delta p_{main}||=\sqrt{\Delta u^{2}+\Delta v^{2}}$, to test the iteration efficiency of proposed seed point generation method. In our experiments, the optimized ZNSSD is converted to zero-mean normalized cross-correlation (ZNCC) to quantify the correlation more straightforward [35]. If the optimized ZNCC is larger than 0.85 and the number of iterations is less than 20, the DIC is considered to be successful.

To evaluate how accurate initial parameters can be estimated by the proposed seed point generation method, experiment results on a plane surface (P), a step surface (S), a cylinder surface (C), and a freeform surface (F) are summarized in Table 1. ${\bar{n}}_{itor}$ is the average number of iterations of the successful seed points. $N_{tri}$ is the number of triangle pairs, $N_{suc}$ is the number of successful seed points generated from the triangle pairs. $R_{suc}$ is the success rate, in other words, the ratio of $N_{suc}$ to $N_{tri}$. The shift vector, (du,dv), of the initial parameters and optimized parameters of u and v is computed for every generated seed point. Then, the root mean square (RMS) of the shift values of all successful seed points is computed.

Two groups of comparison data are listed in Table 1, the convergence criterion for Δp in the two groups is $||\Delta p_{main}||<0.01$. The difference is that the two-step method is used to remove the wrong matches in the second group before the process of seed point generation. The success rates for the three surfaces are all less than 35% in the first group, while the improvements in the second group are evident. For the plane surface and step surface, the success rates are both very near to 100%. $RMS_{du}$ and $RMS_{dv}$ of the four surfaces are about the same in the two groups, which are all around 0.1 pixels. Note that the number of the successful seed points in the second group is about two to four times than that in the first group.

**Figure 9:**
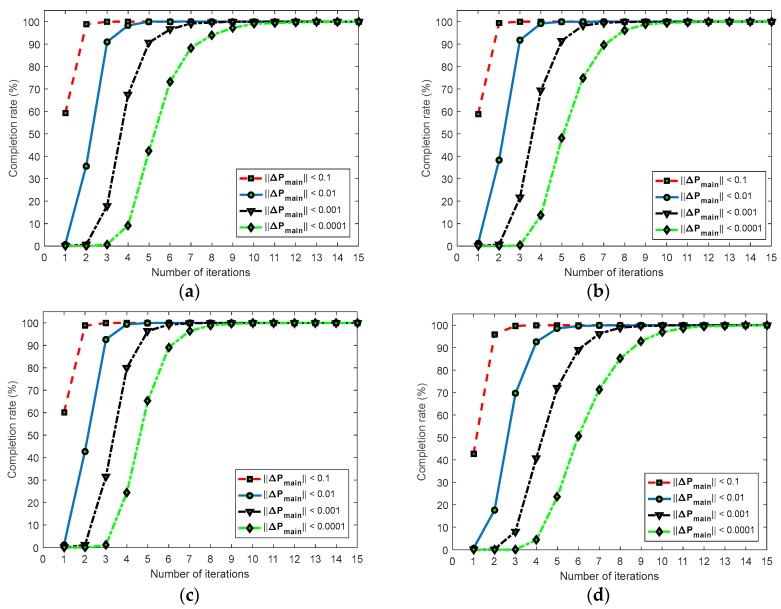

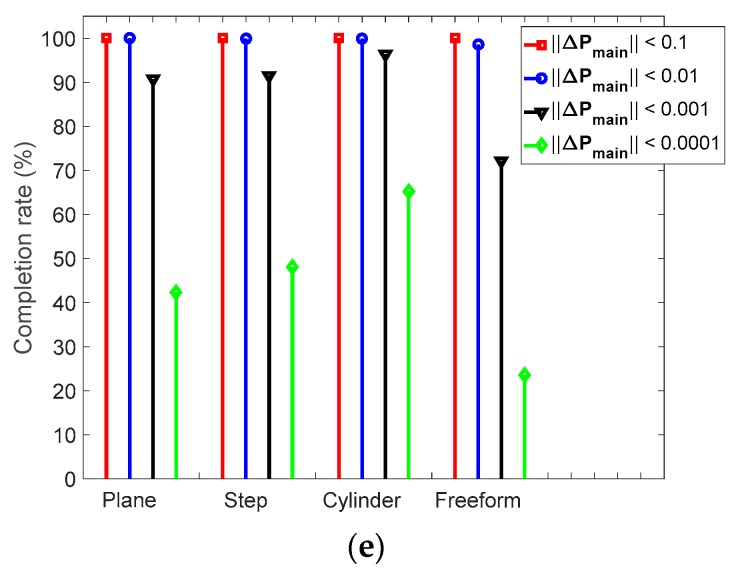
Efficiency evaluation of proposed seed point generation method: (**a**) plane surface; (**b**) step surface; (**c**) cylinder surface; (**d**) freeform surface; and (**e**) completion rate after five iterations.

Furthermore, the efficiency of proposed seed point generation method is tested with different convergence criteria for Δp. As shown in Figure 9, four different criteria for Δp, i.e., $||\Delta p_{main}||<0.1,\;0.01,\;0.001,\;\mathrm{and}\;0.0001$, are used.

Figure 9a–d show the completion rate of the seed points versus the number of iterations of plane surface, step surface, cylinder, and freeform surface, respectively. The completion rate here is the ratio of the number of finished seed points at some iteration to the total number of successful seed points. If $||\Delta p_{main}||<0.1$, more than 40% seed points can be generated by only one iteration. If $||\Delta p_{main}||<0.01$, more than 90% seed points can be generated after four iterations. If $||\Delta p_{main}||<0.001$, the completion rate reaches very near to 100% after seven iterations for all four surfaces. If $||\Delta p_{main}||<0.0001$, it requires more than 10 iterations to reach a 100% completion rate. Note that in Table 1, the numbers of coarse-matched triangle pairs after removing the wrong matches of the three surfaces have large differences from each other. The efficiency of the seed point generation of each surface is also different from other ones because smaller triangle pairs can estimate more accurate initial parameters than larger triangle pairs. Figure 9d shows the completion rate after five iterations of each surface with different criteria. The difference of the convergence speed of $||\Delta p_{main}||<0.1$ and $||\Delta p_{main}||<0.01$ is much smaller than that of $||\Delta p_{main}||<0.01$ and $||\Delta p_{main}||<0.001$. Therefore, $||\Delta p_{main}||<0.01$ is chosen for our measurement, which is also the highly recommended convergence criterion in Pan’s paper [17].

### 4.3. Precision Evaluation

**Figure 10:**
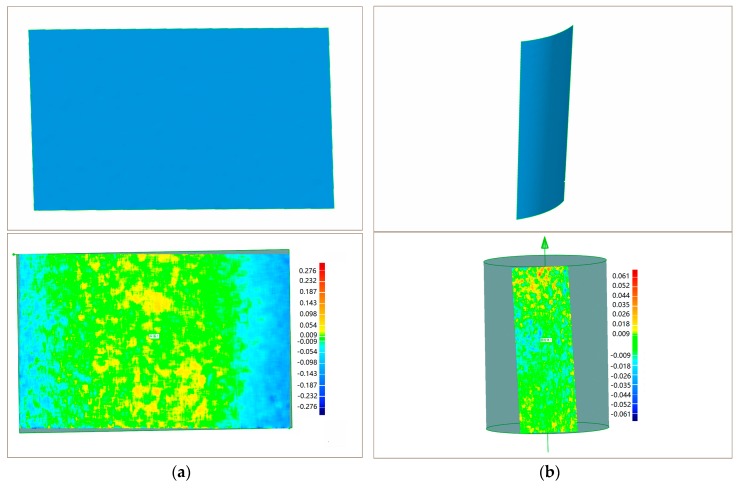
3D shape measurement result and error distribution map: (**a**) plane surface; and (**b**) cylinder surface.

The plane surface and cylinder surface are chosen as standard surfaces to evaluate the measuring precision of the proposed system. The plane surface is made of ceramic and polished to have an accuracy under 0.01 mm. The cylinder surface is the surface of a polish rod, the machining diameter of which is 80 mm with a tolerance of −5~0 mm. The two surfaces are measured on a Coordinate Measuring Machining (CMM (2 + (L/350) µm. Manufactured by Thome Präzision GmbH, Messel, Germany. Supplied by THOME China, Minhang District, Shanghai, China)). Figure 10 shows the 3D shape measurement results and corresponding fitting error distribution maps of the plane surface and cylinder surface. The 3D data from the CMM and proposed measurement system (PMS) are fitted into a plane and cylinder by the least squares method, respectively. The comparison data are listed in Table 2, i.e., point number (PN), negative maximum (NM), positive maximum (PM), standard deviation (SD), and diameter (D).

**Figure 11:**
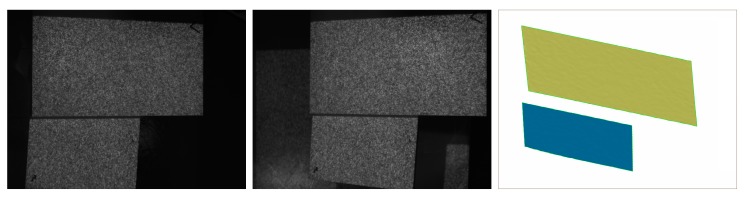

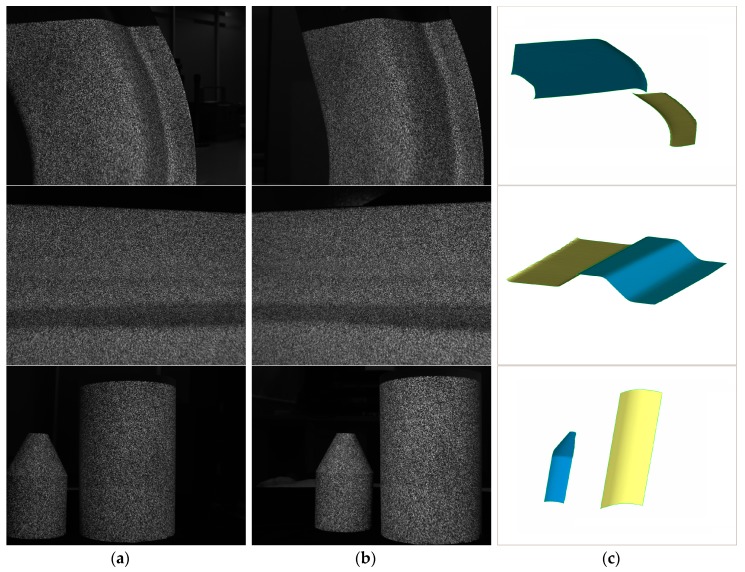
Real objects measurement: (**a**) left image; (**b**) right image; and (**c**) 3D shape.

The CMM measurement results show that the plane surface and cylinder are accurate enough to evaluate the measuring precision of PMS. The standard deviation of plane fitting and cylinder fitting are 0.038 mm, and 0.009 mm, respectively. The diameter of the cylinder measured by CMM and PMS has a difference of 0.041 mm. The above results indicate a high measuring precision of the proposed method. Therefore, the choice of $||\Delta p_{main}||<0.01$ is proper for the proposed system. $||\Delta p_{main}||<0.001$ and $||\Delta p_{main}||<0.0001$ can be used to for higher precision measurement if the hardware of other system allows. More real object measurement tests are shown in Figure 11. The proposed system is effective to measure discontinuous surface and separate objects.

## 5. Conclusions

In this paper, we proposed an accurate seed point generation and efficient background segmentation method for single-shot 3D shape measurement using speckle projection. Firstly, SIFT-based feature matching and two-dimensional triangulation are combined to obtain a coarse matched triangle set. In order to improve the efficiency of seed point generation, a two-step method is proposed to eliminate the wrong matches. Then, a seed point set is obtained from the coarse matched triangle set by DIC with the IC-GN algorithm. Additionally, unnecessary calculations for background pixels are avoided with the proposed background segmentation method. Finally, dense correspondences can be obtained automatically in the ROI by seed point propagation.

Experimental results confirmed the validity of the proposed method. The average segmentation time for image with a resolution of 1280 × 960 pixels is 240 milliseconds. The success rate is evidently improved after removing the wrong matches. More than 90% of seed points can be generated after four iterations for all the tested measuring objects with the convergence criterion of $||\Delta p_{main}||<0.01$. The standard deviations of plane fitting and cylinder fitting are 0.038 mm and 0.009 mm, respectively.

In summary, we hope the proposed method can help broaden the applications in the stereo 3D shape measurement field. In future work, we plan to solve for the measurement limitation on complex surfaces by introducing a second-order DIC. We also intend to find a proper method to improve the background segmentation accuracy by introducing a weighted subset.

## Figures and Tables

**Table 1:** Comparison of seed point generation without and with proposed two-step method for removing wrong matches (unit of RMS: pixel).

	Without Removal						With Removal					
	$RMS_{du}$	$RMS_{dv}$	${\bar{n}}_{itor}$	$N_{tri}$	$N_{suc}$	$R_{suc}$	$RMS_{du}$	$RMS_{dv}$	${\bar{n}}_{itor}$	$N_{tri}$	$N_{suc}$	$R_{suc}$
P	0.1070	0.0995	2.6	1448	322	22.2%	0.1163	0.0988	2.7	758	756	99.7%
S	0.0911	0.0945	2.6	3964	1061	26.8%	0.1022	0.0988	2.7	2310	2272	98.4%
C	0.0952	0.0913	2.6	8709	2715	31.2%	0.0979	0.0849	2.6	6359	4697	73.9%
F	0.1239	0.1043	3.1	38,832	3146	8.1%	0.1355	0.1017	3.2	14,096	12,205	86.6%

**Table 2:** Comparisons of measurement results by CMM and PMS (unit: mm).

	PN		NM		PM		SD		D	
	CMM	PMS	CMM	PMS	CMM	PMS	CMM	PMS	CMM	PMS
Plane	15	491,347	−0.004	−0.276	0.003	0.251	0.001	0.038		
Cylinder	44	253,580	−0.008	−0.040	0.011	0.061	0.004	0.009	79.952	79.911
